# A cross-sectional investigation of the health needs of asylum seekers in a refugee clinic in Germany

**DOI:** 10.1186/s12875-018-0758-x

**Published:** 2018-05-16

**Authors:** Laura F. Goodman, Guy W. Jensen, Joseph M. Galante, Diana L. Farmer, Stephanie Taché

**Affiliations:** 1 Department of Surgery, University of California Davis Health, 2315 Stockton Blvd, OP 512, Sacramento, CA 95817 USA; 2 General Medicine Division, MK3, Technical University Dresden, Carus School of Medicine, Fetscherstrasse 74, 01307 Dresden, Germany

**Keywords:** Asylum seeker, Refugee, Public health, Health care, Health systems, Epidemiology

## Abstract

**Background:**

Over one million asylum seekers were registered in Germany in 2016, most from Syria and Afghanistan. The Refugee Convention guarantees access to healthcare, however delivery mechanisms remain heterogeneous. There is an urgent need for more data describing the health conditions of asylum seekers to guide best practices for healthcare delivery. In this study, we describe the state of health of asylum seekers presenting to a multi-specialty primary care refugee clinic.

**Methods:**

Demographic and medical diagnosis data were extracted from the electronic medical records of patients seen at the ambulatory refugee clinic in Dresden, Germany between 15 September 2015 and 31 December 2016. Data were de-identified and analyzed using Stata version 14.0.

**Results:**

Two-thousand-seven-hundred and fifty-three individual patients were seen in the clinic. Of these, 2232 (81.1%) were insured by the state indicating arrival within the last 3 months. The median age was 25, interquartile range 16–34. Only 786 (28.6%) were female, while 1967 (71.5%) were male. The most frequent diagnoses were respiratory (17.4%), followed by miscellaneous symptoms and otherwise not classified ailments (R series, 14.1%), infection (10.8%), musculoskeletal or connective tissue (9.3%), gastrointestinal (6.8%), injury (5.9%), and mental or behavioral (5.1%) categories.

**Conclusions:**

This study illustrates the diverse medical conditions that affect the asylum seeker population. Asylum seekers in our study group did not have a high burden of communicable diseases, however several warranted additional screening and treatment, including for tuberculosis and scabies. Respiratory illnesses were more common amongst newly arrived refugees. Trauma-related mental health disorders comprised half of mental health diagnoses.

**Electronic supplementary material:**

The online version of this article (10.1186/s12875-018-0758-x) contains supplementary material, which is available to authorized users.

## Background

There were 65.5 million displaced persons worldwide in 2016 [1]. Approximately 1.3 million people applied for asylum in Europe in 2016 [2], most fleeing from Syria, Afghanistan and South Sudan [3]. Germany was the primary destination for asylum seekers in 2016, receiving 722,400 out of the 2.8 million applications worldwide [3].

Asylum seekers in Germany are excluded from routine health monitoring systems such that the body of knowledge on refugee health status in Germany is limited [4]. However, a small number of studies describe a broad range of diagnoses [5, 6]. A systematic review of studies on health status and medical care among refugees and asylum seekers in Germany identified three publications not using a disease-based approach for comparison [4]. The remaining available studies focused on specific specialties such as infectious diseases, psychiatry, or special populations such as minors. A recent study of health conditions amongst asylum seekers at a camp in Brussels found that upper airway infections were the most common diagnoses, followed by dental caries, and skin infection [5]. A 2014 review of non-communicable diseases (NCDs) among urban refugees in developing countries found the prevalence of NCDs amongst those from the Middle East ranged from 9 to 50% [7]. Another recent review noted that the prevalence of TB in Syria, the country of origin of most asylum seekers in Germany in 2015, was lower than that in several European Union countries [8]. A small pilot study of disease prevalence among youth asylum seekers in Germany found that over half had infections, nearly half of which were *Helicobacter pylori* [9].

**Fig. 1:**
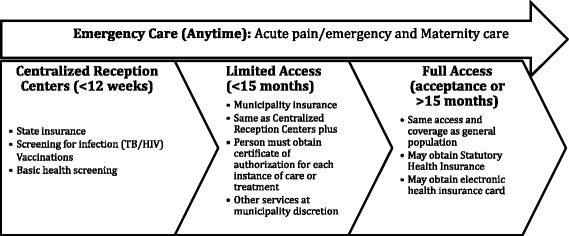
Germany’s asylum seeker healthcare schema. After first arrival, asylum seekers can receive emergency care at any time, and receive screening at centralized reception centers. During this initial period up to 12 weeks, asylum seekers have State insurance. After 3 months, limited access is granted and insurance is provided by the municipality. This lasts up to 15 months, after which full access is possible. Permission to base this figure on a similar one previously published was granted by the author S. Bauhoff [22]

In response to the influx of asylum seekers in 2015, Germany mounted a nationwide response to secure basic needs including medical care for this population [10] (see Fig. 1). In this study, *asylum seeker* refers to both those who have received formal asylum (refugees) and those who have applied for refugee status. The state of Saxony received approximately 24,000 asylum-seekers in the second half of 2015 [11]. In response, three ambulatory refugee clinics were created in Saxony. The first was opened in September 2015 in Dresden [12, 13].

Our study describes the medical diagnoses among asylum seekers in one clinic in Dresden. Through this study, we aim to add to the nascent epidemiological data characterizing this population.

## Methods

### Materials, methods, and setting

Implemented as a partnership between the city of Dresden, the state of Saxony, and the regulating union for outpatient care (*Kassenärztliche Vereinigung Sachsen* or KVS), the refugee clinic functioned as a first medical triage and care point for asylum seekers in Dresden. It provided on-site diagnostic and treatment services in general medicine, pediatrics, and psychiatry.

### Data source

Data for all patients seen in the asylum-seeker clinic in the period 14 September 2015–31 December 2015 were extracted from the electronic medical record (Turbomed) and de-identified by the staff at KVS. Age, gender, and insurance category, were merged with diagnosis(es) with International Classification of Disease-10 (ICD-10) codes and clinic visit code. Unlinked data on country of origin of the study’s source population of asylum seekers in Saxony were available from the State of Saxony [11]. The study was deemed exempt by the University of California Davis Institutional Review Board (2 February 2016, IRB # 852673–1) and approved by the Kassenärztliche Vereinigung Sachsen Ethics Committee in Dresden. Patient consent was not obtained, as the data were de-identified.

### Data analysis

Only patients with demographic, insurance, and diagnosis data were included in the analyses. Private insurance holders were excluded, as were patients missing insurance data, diagnosis data, or those who were unable to be matched between the insurance and diagnosis databases. The last recorded insurance status was used in the demographic analyses for patients who changed insurance during the study period. Many patients had more than one ICD-10 diagnosis per visit and all the ICD-10 codes were included in analyses. When a new diagnosis was made as part of a patient encounter, providers had the option of specifying whether it was suspected, confirmed, or ruled out. We thus categorized the ICD-10 codes by validity as either suspected, confirmed, or ruled out. Ruled out ICD-10 codes were excluded except for UTI’s.

Age and gender characteristics of each diagnosis group were compared, as was the insurance composition of each diagnosis group, using student’s t-test, Mann-Whitney U test, chi-squared or Fisher’s exact tests, as appropriate. The additional category of chronic diseases was created, to include non-communicable diseases and was analyzed as per the other diagnostic groups. Ninety-five percent confidence intervals were calculated. All analyses were completed using Stata 14 [14].

## Results

After excluding 43 private insurance holders, 2753 non-duplicated patients were seen in the clinic between 14 September 2015 and 31 December 2015. There were 6423 ICD-10 codes. After eliminating missing diagnosis data, insurance data, and non-asylum seeking status, 6361 ICD-10 codes were used for the analysis. There were 4291 unique clinic visits. Ninety ICD-10 codes that were listed but were clinically ruled out were excluded from analyses. However, those patients with ruled out diagnoses were included in the demographic summaries.

### Demographics

Ages ranged from 1 to 87 years, with a mean of 25.3 and standard deviation of 14.9 years, median 25, interquartile range 16–34 (see Additional file 1). Of the 2753 included patients, 1967 (71.5%) were male, and 786 (28.5%) were female. There were 1949 patients (70.8%) over the age of 18 years and 804 (29.2%) aged 18 or under. Only 29 (1.1%) were over 65 years of age.

The State Directorate of Saxony’s data indicate that 42.5% were from Syria, 14.6% from Afghanistan, 11.7% from Iraq, and the remainder as illustrated in Additional file 2 [11].

Most patients were insured by the state of Saxony (2232 or 81.1%), a minority insured by the city of Dresden (472, 17.1%), and fewer by youth or other asylum-seeker insurance (49, 1.8%, see Table 1). Females comprised 794 or 28.4% of the overall of the clinic population; a greater number were insured by the state (*n* = 697, comprising 31.2% of those insured by the state) than by the city (*n* = 75, 16.9% of those insured by the city). Unaccompanied minors were insured through the Youth Ministry and represented 1.6% of the clinic population.

**Table 1:** Patient demographics and insurance status

	Total (*n* = 2753)	State Insurance (*n* = 2232, 81.1%)	Combined Other: All Non-State (*n* = 521, 18.9%)	City of Dresden (*n* = 472, 17.1%)	Unaccomp-anied Youth (*n* = 45, 1.6%)	Other (n = 4, 0.15%)	*p*-value, State vs. Non-State Insurance
Age in years (mean (SD))	25.3 (14.9)	25.2 (15.6)	26.1 (11.9)	27.0 (12.0)	16.0 (2.94)	30.0 (10.0)	0.0476
Male (n, %)	1967 (71.4)	1535 (68.8)	432 (82.9)	385 (81.6)	43 (95.6)	4 (100)	< 0.001
Minors (n, %)	804 (29.2)	683 (30.6)	121 (23.2)	76 (16.1)	45 (100)	0	0.001
Elderly (n, %)	29 (1.1)	28 (1.3)	1 (0.2)	1 (0.2)	0	1	0.017

Patient mean age, number and proportion with male gender, number and proportion minors, and number and proportion elderly, in entire population and by insurance status. State insurance is granted upon first arrival in Germany. Other insurance statuses require greater than 3 months in Germany to obtain, indicating these patients were in the country over 3 months. The last known insurance status of patients seen more than once was used. Mann-Whitney U test was used to obtain *p* values for continuous outcomes, and Pearson’s chi-squared test for binary outcomes, comparing state to non-state insurance, 1-sided Fisher’s exact chi-squared was used for the elderly category

Most diagnoses occurred in recently arrived refugee/state-insured group (82.4%, *n* = 5170, 95% CI 81.5–83.3). Meanwhile, 16.0% (*n* = 1101) of diagnoses occurred in those residing in Germany over 3 months and had other insurance.

### Diagnostic groups and diagnoses

There were 6271 diagnoses comprising 683 different diagnostic codes. The most frequent categories were respiratory (J series, *n* = 1090), miscellaneous symptoms and otherwise not classified ailments (R series, *n* = 881), infection (A and B series, *n* = 678), musculoskeletal or connective tissue (M series, *n* = 583), gastrointestinal (K series, *n* = 427), injury or poisoning (S and T series, *n* = 372), and mental or behavioral (F series, *n* = 322, see Table 2 and Additional file 1: Figure S1).

**Table 2:** Patient demographics by diagnosis category

Diagnosis category (ICD-10 Group)	Diagnoses (n, %)	Patients (n)	Age (mean years ± SD)	Age 95% CI	Proportion Male (95% CI)	*P*-Value Male	Proportion Minors (95% CI)	*P*-Value Minors
Total	6271 (100)	2753	25.3 ± 14.9	24.8–25.9	0.71 (0.70–0.73)	–	0.29 (0.28–0.31)	–
Respiratory (J)	1090 (17.4)	849	21.1 ± 15.9	20.0–22.1	0.68 (0.64–0.71)	0.029	0.43 (0.4–0.47)	< 0.001
Miscellaneous Abnormalities (R)	881 (14.1)	667	26.1 ± 14.8	25.0–27.3	0.69 (0.66–0.73)	0.215	0.26 (0.22–0.29)	0.057
Infection (A, B)	678 (10.8)	546	18.9 ± 14.3	17.7–20.1	0.71 (0.67–0.75)	0.925	0.47 (0.43–0.51)	< 0.001
Musculoskeletal/ Connective Tissues (M)	583 (9.3)	384	32.6 ± 12.9	31.3–33.8	0.73 (0.69–0.78)	0.415	0.083 (0.06–0.12)	< 0.001
Digestive (K)	427 (6.8)	314	27.8 ± 14.6	26.2–29.4	0.75 (0.70–0.80)	0.161	0.21 (0.17–0.26)	0.002
Injury/ Poisoning (S, T)	372 (5.9)	261	26.1 ± 12.4	24.5–27.6	0.84 (0.79–0.88)	< 0.001	0.23 (0.18–0.29)	0.034
Mental/ Behavioral (F)	322 (5.1)	172	29.4 ± 12.2	27.5–31.2	0.65 (0.58–0.72)	0.077	0.15 (0.1–0.21)	< 0.001

The diagnosis categories included patients with ruled out diagnoses in those categories, and the categories are not mutually exclusive. Tests of proportion (chi-squared) were used to obtain *p*-values. Not shown are categories comprising fewer than 5 % of diagnoses

### Respiratory (J00-J98.9): *n* = 1090 diagnoses, *n* = 849 patients

Respiratory diagnoses affected over one-third of the clinic population. Of those diagnoses, the most frequent were acute upper respiratory infection (*n* = 452), acute tonsillitis, and bronchitis. Asthma comprised less than 3 % of diagnoses.

**Fig. 2:**
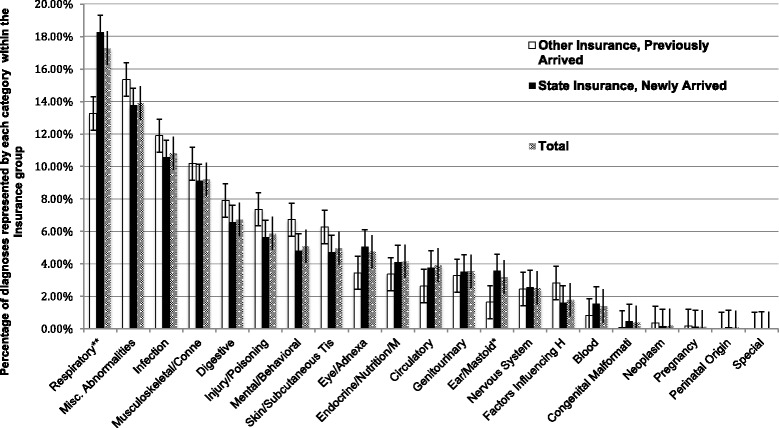
Diagnostic categories by insurance (arrival) status. Comparing the proportion by insurance status state vs. other **p < 0.001 *p = 0.0043, other differences were not significant. Standard error bars are shown

Respiratory diagnoses occurred more often in female, in younger and recently arrived patients when compared with the clinic population as a whole. Male patients comprised 67.5% of those with respiratory diagnoses (95% CI 64.3–70.6), compared with 71.4% of the clinic population (95% CI 69.7–73.1, *p* = 0.029, see Table 2.) The mean age was 21.1 (95% CI 20.0–22.1), significantly lower than the overall clinic population (mean 25.3, 95% CI 24.8–25.9), and 43.3% of patients with respiratory diagnoses were minors (95% CI 40.0–46.7). Of the respiratory patients, 87.3% were recently arrived and insured by the state (95% CI 84.9–89.4), compared with 81% of the total clinic population (*p* < 0.001, see Fig. 2 and Additional file 3: Figure S3).

### Miscellaneous signs and symptoms (R00-R94.2): *n* = 881 diagnoses, *n* = 667 patients

The most common symptom was other and unspecified abdominal pain (R10.4, *n* = 130, 14.8%), followed by headache (R51, *n* = 121, 13.7%), cough (*n* = 64, 7.26%), pain in throat (*n* = 44, 4.99%), unspecified pain (*n* = 44, 4.99%), and heartburn (*n* = 34, 3.86%). The remaining symptoms or signs ranged from dysuria to epistaxis, jaundice, and malaise, to halitosis.

Miscellaneous diagnoses trended mostly male (457, 68.8%), not significantly different from the clinic population. The mean age was 25.0, not significantly different from the clinic population. The proportion of minors (25.5%) did not reach statistical significance (*p* = 0.0568). The proportion of newly arrived was not statistically different from the proportion of the entire population affected (*p* = 1.0).

### Infection (A03.9-B99): *n* = 678 diagnoses, *n* = 546 patients

Unspecified viral infections were most frequent infectious diagnosis (B34.9, *n* = 160, 23.6%), followed by gastroenteritis/colitis (A09.9, *n* = 114, 16.8%), scabies (B86, *n* = 96, 14.2%), unspecified mycosis (B49, *n* = 33, 4.87%), and respiratory tuberculosis (A16.9, *n* = 26, 3.83%). There were two diagnoses of tuberculous pleurisy (A16.5), one diagnosis of mycoplasma pneumonia (B96.0). There were no HIV diagnoses, six acute hepatitis B infections, five chronic hepatitis B infections, and nine chronic hepatitis C diagnoses. Six sexually transmitted infections were diagnosed, including gonorrhea, herpes, and chlamydia. Superficial infestations accounted for 204 diagnoses or 30.1% skin diagnoses, including scabies, superficial mycosis, viral warts, candidiasis of skin and nail, pediculosis, tinea corporis, and tinea pedis. Minors most commonly presented with unspecified viral infections (B34.9, *n* = 104, 31.9%), followed by gastroenteritis and colitis (A09.9, *n* = 77, 23.6%), and scabies (B86, *n* = 23, 7.06%). Of the 18 patients with suspected TB, ten were minors.

Patients with infections trended male (384, 71.2%), no different from the entire population. Those with infections had a low mean age: 18.9 (95% CI 17.7–20.1). A significantly higher proportion of those with infections were minors as compared with the entire clinic population (46.9%, 95 CI 42.7–51.1%, *p* < 0.001). Insurance type did not correlate with the proportion of those with infectious diseases (see Table 2).

Musculoskeletal (M06.9-M94.0): *n* = 583 diagnoses, *n* = 384 patients.

The most common musculoskeletal diagnoses were pain in joint (M25.5, *n* = 112, 19.2%), pain in limb (M79.6, *n* = 93, 16.0%), and unspecified dorsalgia (M534.9, *n* = 92, 15.8%). Less common were low back pain (M54.5, n = 38, 6.52%), followed by radiculopathy (M54.1, *n* = 36, 6.17%), myalgia (M79.1, *n* = 16, 2.74%), unspecified arthrosis, and arthritis of rheumatoid and unspecified types. There were nine osteomyelitis diagnoses (M86.9), six unspecified osteonecrosis (M87.9), and four diagnoses of gonarthrosis (M17.9).

Musculoskeletal diagnoses trended male (73.4%), not different from the population. The mean age was 32.6 (95% CI 31.3–33.8), significantly older than the whole clinic population. Only 8.30% of the group with musculoskeletal diagnoses was minor (95% CI 5.90–11.6%) less than the total group (29.2, 95% CI 27.5–30.9%, *p* < 0.001). Most were recently arrived and insured by the state (78.9%), not significantly different from the source clinic population.

### Digestive or gastrointestinal (K00-K92.9): *n* = 427 diagnoses, *n* = 314 patients

Gastrointestinal diagnoses included constipation (K59.0, *n* = 95, 22.3%), gastritis unspecified (K29.7, *n* = 65, 15.2%), and other specified disorders of teeth (K08.8, *n* = 53, 12.4%), followed by hemorrhoids (K64.9, *n* = 44, 10.3%), inguinal hernia (K40.9, *n* = 14, 3.28%), anal fissure (K60.2, *n* = 10, 2.34%), gastrointestinal reflux disease (K21.9, *n* = 9, 2.11%), and melena (K92.1, *n* = 8, 1.87%).

Gastrointestinal diagnoses were mostly among males (75.2%), reflecting the population. The mean age of those with gastrointestinal disorders was 27.8 years (95% CI 26.2–29.4), significantly older than the mean of the clinic population (25.3 years, 95% CI 24.8–25.9). The proportion of minors with a gastrointestinal diagnosis was significantly lower than published in other studies (20.7, 95% CI 16.6–25.5%, *p* = 0.0015) [9]. The proportion of newly arrived (78.9%) was not significantly different from the source population.

### Injuries and poisoning (S00-T88.7): *n* = 372 diagnoses, *n* = 261 patients

The most common injury category was unspecified (T14.9, *n* = 77, 20.7%), followed by post-traumatic wound infection (T79.3, *n* = 24, 6.45%), and fracture of unspecified body region (T14.2, *n* = 22, 5.91%). There were 154 fracture diagnoses comprising 41.4% of the injury diagnoses. Open wounds (T14.0, *n* = 16, 4.3%), and superficial injury of unspecified body part (T14.1, n = 16, 4.3%) were next most numerous. Sprain and strain of ankle (*n* = 10, 2.69%) and dislocation, sprain, and strain of other body part (n = 10, 2.69%) were less common.

Injury diagnoses were more male (83.9%, 95 CI 78.9–87.9%) than the general clinic population (71.4, 95% CI 69.7–73.1, *p* < 0.001). The mean age was 26.1 (95% CI 24.5–27.6), while the overall clinic population mean was 25.3. More of the injured patients were minors (32, 95% CI 18.3–28.5%, *p* = 0.034). Similar to the entire clinic population, most injured patients were newly arrived (77.4, 95% CI 72.3–82.5%, *p* = 0.147).

### Mental behavioral (F00-F99): *n* = 322 diagnoses, *n* = 172 patients

The most common mental health diagnosis was post-traumatic stress disorder (PTSD F43.1, *n* = 57, 17.7% of mental health diagnoses), followed by unspecified depressive episode (F32.9, *n* = 41, 12.7%), adjustment disorders (F43.2, *n* = 34, 10.6%), somatization disorder (F45.0, *n* = 22, 6.83%), and unspecified somatoform disorder (F45.9, *n* = 18, 5.59%). The total diagnosis count of PTSD, adjustment, stress reaction, and somatoform disorders (F43.0-F45.9) combined was 153 or 47.5% of the category. Mental or behavioral disorders related to substance use, dependence, or abuse (F10.1-F19.2), accounted for 18 diagnoses.

A non-significantly lower percentage of patients with mental or behavioral diagnoses were male in comparison to the rest of the clinic population (65.1, 95% CI 57.8–71.8%, *p* = 0.0772). The mean age of 29.4 (95% CI 11.8–21.9) is not significantly different from the clinic population. However, only 14.5% of patients with these diagnoses were minors (95% CI 10.0–20.6), compared with 29.2% overall (*p* < 0.001). Most of the patients were newly arrived with no difference from the clinic population (80.2, 95% CI 73.6–85.5%, *p* = 0.77).

### Skin or subcutaneous (L00-L99): *n* = 313 diagnoses, *n* = 249 patients

The most common skin diagnoses were unspecified dermatitis (L30.9, *n* = 59, 18.9%), abscess, furuncle or carbuncle (L02.9, *n* = 30, 9.58%), unspecified acne (L70.9, *n* = 30, 9.58%), psoriasis (L40.9, *n* = 26, 8.31%), unspecified pruritis (L29.9, *n* = 22, 7.03%), local infection (L08.9, *n* = 15, 4.79%), and unspecified follicular disorder (L73.9, *n* = 14, 4.47%).

Similarly to the general clinic population, patients with skin or subcutaneous diagnoses trended male (75.9%, 95 CI 70.2–80.8%, *p* = 0.131), but had a lower mean age at 22.3 years (95% CI 20.6–24.0). Minors comprised 36.5% (95% CI 30.8–42.7%) of the patients compared with 29.2% overall (*p* = 0.0159). There was no significant difference in the proportion of newly arrived compared with the clinic population (79.9, 95% CI 74.5–84.4%).

### Genitourinary (N00-N099): *n* = 218 diagnoses, *n* = 212 patients

Urinary tract infection (UTI) was the most common genitourinary diagnosis, occurring 42 times (19.3%), followed by cystitis, unspecified (N30.9, *n* = 22, 10.1%), acute vaginitis (*N* = 76.0, n = 21, 9.63%), and calculus of kidney (N20.0, *n* = 18, 8.26%). UTI was ruled out in 35 cases.

Genitourinary diagnoses were mostly diagnosed in female patients; only 43.4% were in males (95% CI 36.9–50.1%, *p* < 0.001). The mean age was not significantly different from the clinic population at 26.2 (95% CI 24.3–28.2). Minors made up 25.0% of this group, not significantly different from the general clinic population. Most (83%) were newly arrived and insured by the state.

### Pregnancy-related (O00-O99, Z33, Z34): *n* = 49 diagnoses, *n* = 40 patients

Of the 786 female patients, there were 40 non-duplicated patients pregnant at the time of data collection (5.09% of female patients). The mean age was 26.0 years (95% CI 24.1–27.8), and five were minors (12.0%, 95 CI 2.30–22.7%, *p* = 0.0208). Most (72.5%) were recently arrived and insured by the state, not significantly different from the general clinic population.

### Chronic diseases (see Additional file 4: Table S1 for ICD10 codes included): *n* = 519 diagnoses, *n* = 293 patients

Chronic conditions excluding mental health diagnoses were present in 293 patients. The most common diagnoses were hypertension (I10.9, *n* = 124), unspecified diabetes mellitus (E14.9, *n* = 77), unspecified epilepsy (G40.0, *n* = 54), and type 2 diabetes mellitus without complication (E = 11.9, *n* = 53). Other common diagnoses included chronic obstructive pulmonary disease (*n* = 31), thalassemia (*n* = 30), and asthma (*n* = 28).

The mean age was significantly higher than the source population (33.6 years, 95% CI 31.6–35.5). There was a significantly lower proportion of minors (17.1, 95% CI 13.2–21.8%, *p* < 0.001). This group was 66.2% (95% CI 60.1–71.4%) male, not significantly different from the clinic population (*p* = 0.0625). There was a non-significantly higher proportion of newly arrived patients in this group (85.0, 95% CI 80.4–88.6%, *p* = 0.102).

## Discussion

### Epidemiological findings

#### Representativeness of population

The age and sex of our population were consistent with data on newly arriving refugees from the state of Saxony (Additional file 5: Figure S4) [11]. Because our database did not capture nationality or country of origin, we can only surmise that the patients were similar to the asylum seekers in the state of Saxony at the time, with the majority from Syria, followed by Afghanistan and Iraq [11]. The most requested languages for assistance in the clinic were Arabic, Farsi, and Dari. Eighty percent of patients lived in centers located in and around the city of Dresden, and because our clinic was the only point of first care for refugees in the area, we postulate that most of the ambulatory non-emergency medical care they received occurred in our clinic.

### Disease burden profile

In spite of the common perception of an association between migration and the importation of infectious diseases we did not find such an association in our study as seen in the low number of transmissible infections.

Asylum seekers within 3 months of arrival in Germany, as identified by insurance status, were significantly more likely to have a respiratory diagnosis, mostly infectious, when compared with asylum seekers with three or more months of residence (*p* < 0.001). The preponderance of respiratory infections is consistent with recent studies in Belgium, Germany, and Switzerland [8, 15, 16]. More frequent respiratory diagnoses among new arrivals are likely attributable to conditions in Dresden’s reception centers, which often had open sleeping arrangements and minimal temperature control. Exposure and stress during the flight to Germany may also have contributed. In our study, ear and mastoid diagnosis numbers may also be related to flight circumstances. The proportion of minors with a gastrointestinal diagnosis (20.7%) was significantly lower than that published in other studies (50%) [9].

Non-Communicable Diseases (NCD) represent a newly recognized challenge in refugee operations [17]. However, this population had fewer NCD diagnoses (10.8%), as compared with the age-matched (19–29 years) population 16.3% (95% CI 14.4–18.4%) and the general population 38.8% (95% CI 37.9–39.7%) in Germany [18]. The lower level of NCDs documented in our clinic may either be due to self-selection at the time of immigration, decreased care-seeking among patients with chronic diseases, or even missed diagnoses. There may also be a change in the composition of the patient population over time, as the initial migrations during the summer of 2015 were primarily by foot, whereas in the fall and winter months, more asylum seekers travelled by bus and train [19]. Except for certain documented cases of asylum seekers with mobility problems traveling the Balkan corridor during the summer 2015, most people seeking refuge had to be physically fit and healthy enough to endure the difficult conditions associated with the journey. This changed in late Fall and early Winter 2015, when buses or trains were made available to shuttle people across countries along the Balkan route [20]. This possibly enabled a higher proportion of people with physical disabilities or physically limiting NCDs to undertake the journey. Our study sample did not demonstrate higher rates of NCDs among longer-established refugees compared with newly arrived refugees.

One diagnostic category inconsistent with prior literature is the higher level of eye diagnoses [21]. Soon after the clinic opened, a major optical firm donated 160 vouchers for new glasses for qualified patients and may account for the overrepresentation of eye-related diagnoses in our study.

The mental health prevalence documented in refugee populations in Germany is variable, ranging from 6.65–76.66% for institution-based studies such as ours, to 16.36–54.90% in population based studies [4]. In comparison to these studies, the mental health prevalence in our sample appears low. This underrepresentation of mental health prevalence in our sample may be explained because 1) mental health disorders often presented as non-specific pain or psychosomatic complaints to the primary care physicians and 2) the psychiatrists, who carried out the majority of mental health diagnoses in our clinic, had limited patient appointment availability. The reliability and validity of the mental health diagnoses in our study are thus difficult to ascertain.

### Limitations

The short time frame of available data limits this study. Another limitation is the absence of patient demographic data. Data not directly linked to medical care could not be collected due to German law. Another limitation is the use of ICD-10 diagnostic categories as a proxy of disease prevalence: this prevents weighing diagnoses by order of importance in patients receiving multiple diagnoses, which leads to a low-resolution epidemiological topography.

## Conclusions

This descriptive epidemiological study confirms previous findings of disease prevalence in newly arrived asylum seekers. Common health problems comprise communicable diseases including upper respiratory, gastrointestinal and skin infections, injuries and wounds, as well as the psychiatric sequelae of trauma. Aside from upper respiratory infections, no significant differences in disease profile between newly arrived and longer settled refugees were found.

### Keypoints

- Our findings are similar to previously published studies from Germany and Belgium documenting how the first migratory wave consists of mostly male, relatively young and relatively healthy individuals.
- Asylum seekers in Dresden did not bear a high burden of communicable diseases, but several warranted additional screening and treatment, including for tuberculosis and scabies.
- Respiratory illnesses were more common amongst those who had been in Germany less than 3 months compared with those residing longer, and may indicate a need for improved care and conditions immediately upon arrival and during flight.
- Nearly half of the mental health disorders were trauma-related (PTSD, adjustment, stress reaction, and somatoform disorders).
- The prevalence of chronic disease in this study was relatively low.
- This study helps determine which primary care based screening and treatment interventions may be most appropriate for a population of asylum seekers in Europe.

## Additional files

Additional file 1: **Figure S1.** Patient age and gender distribution. All asylum seeker patients seen in the period 14 September to 31 December 2015 are plotted by age and gender, including those with ruled-out diagnoses, by five-year increments. (DOCX 64 kb)

Additional file 2: **Figure S2.** Distribution of ICD-10 diagnosis categories. Number of diagnoses are shown on the Y-axis. (DOCX 60 kb)

Additional file 3: **Figure S3.** Fifteen most common diagnoses. The fifteen most common diagnoses by ICD-10 codes are plotted with the number of diagnoses for each code on the Y-axis. (DOCX 71 kb)

Additional file 4: **Table S1.** Diagnoses included in chronic conditions. (DOCX 95 kb)

Additional file 5: **Figure S4.** Countries of origin of asylum seekers in Saxony 2015. Countries of origin are plotted with percentage of total asylum seekers who arrived in Saxony between 1 January and 31 December 2015. The data is from *Landesdirektion Sachsen*, or the State Directorate of Saxony [11]. (DOCX 77 kb)

## Abbreviations

CI: Confidence interval
ICD-10: International Statistical Classification of Diseases and Related Health Problems, 10th revision
KVS: *Kassenärztliche Vereinigung Sachsen* (regulating body for outpatient care in Saxony)
LDS: *Landesdirektion Sachsen* (Government of Saxony)
NCD: Non-communicable diseases
SD: Standard deviation
TB: Tuberculosis
UTI: Urinary tract infection
WHO: World Health Organization
