# Supplementary material for: A cross-sectional investigation of the health needs of asylum seekers in a refugee clinic in Germany
Source: BMC Fam Pract. 2018 May 16;19:64. doi: 10.1186/s12875-018-0758-x (PMC5956552; doi:10.1186/s12875-018-0758-x)
Supplement: Supplementary file 1 — Figure S1. Patient age and gender distribution. All asylum seeker patients seen in the period 14 September to 31 December 2015 are plotted by age and gender, including those with ruled-out diagnoses, by five-year increments. (DOCX 64 kb) [file 12875_2018_758_MOESM1_ESM.docx]

**Supplement 1** Patient age and gender distribution

Female **Male**
