# Supplementary material for: A cross-sectional investigation of the health needs of asylum seekers in a refugee clinic in Germany
Source: BMC Fam Pract. 2018 May 16;19:64. doi: 10.1186/s12875-018-0758-x (PMC5956552; doi:10.1186/s12875-018-0758-x)
Supplement: Supplementary file 2 — Figure S2. Distribution of ICD-10 diagnosis categories. Number of diagnoses are shown on the Y-axis. (DOCX 60 kb) [file 12875_2018_758_MOESM2_ESM.docx]

**Supplement 2.**  Countries of origin of asylum seekers who arrived in Saxony between 1 January – 31 December 2015
