# Supplementary material for: A cross-sectional investigation of the health needs of asylum seekers in a refugee clinic in Germany
Source: BMC Fam Pract. 2018 May 16;19:64. doi: 10.1186/s12875-018-0758-x (PMC5956552; doi:10.1186/s12875-018-0758-x)
Supplement: Supplementary file 3 — Figure S3. Fifteen most common diagnoses. The fifteen most common diagnoses by ICD-10 codes are plotted with the number of diagnoses for each code on the Y-axis. (DOCX 71 kb) [file 12875_2018_758_MOESM3_ESM.docx]

**Supplement 3** Distribution of diagnostic code categories
