# Supplementary material for: A cross-sectional investigation of the health needs of asylum seekers in a refugee clinic in Germany
Source: BMC Fam Pract. 2018 May 16;19:64. doi: 10.1186/s12875-018-0758-x (PMC5956552; doi:10.1186/s12875-018-0758-x)
Supplement: Supplementary file 4 — Table S1. Diagnoses included in chronic conditions. (DOCX 95 kb) [file 12875_2018_758_MOESM4_ESM.docx]

**Supplement**  Diagnoses included in chronic conditions

| B18.0 Chronic viral hepatitis B with delta agent |
| --- |
| B18.2 Chronic viral hepatitis C |
| D50.9 Iron deficiency anaemia, unspecified |
| D51.9 Vitamin B12 deficiency anaemia |
| D56.9 Thalassaemia, unspecified |
| D57.0 Sickle-cell anaemia with crisis |
| D57.1 Sickle-cell anaemia without crisis |
| E03.9 Hypothyroidism, unspecified |
| E10.4 Type 1 diabetes mellitus: With neurological complication |
| E10.9 Type 1 diabetes mellitus: Without complication |
| E11.4 Type 2 diabetes mellitus: With neurological complication |
| E11.7 Type 2 diabetes mellitus: With multiple complications |
| E11.9 Type 2 diabetes mellitus: Without complication |
| E14.9 Unspecified diabetes mellitus: Without complication |
| E23.0 Hypopituitarism |
| E61.1 Iron deficiency |
| E66.9 Obesity, unspecified |
| E78.0 Pure hypercholesterolaemia |
| E78.5 Hyperlipidaemia, unspecified |
| E78.9 Disorder of lipoprotein metabolism |
| G40.9 Epilepsy, unspecified |
| G47.3 Sleep apnoea |
| H26.9 Cataract, unspecified |
| H40.9 Glaucoma, unspecified |
| I20.9 Angina pectoris, unspecified |
| I25.1 Atherosclerotic heart disease |
| I25.9 Chronic ischaemic heart disease |
| I38 Endocarditis, valve unspecified |
| I44.3 Other and unspecified atrioventricular block |
| I48.9 Atrial fibrillation and atrial flutter |
| I49.3 Ventricular premature depolarization |
| I49.9 Cardiac arrhythmia, unspecified |
| I50.9 Heart failure, unspecified |
| I51.9 Heart disease, unspecified |
| I70.2 Atherosclerosis of arteries of arteries of extremities |
| I73.9 Peripheral vascular disease, unspecified |
| I83.9 Varicose veins of lower extremity |
| I86.1 Scrotal varices |
| I87.2 Venous insufficiency (chronic) |
| J44.8 Other specified chronic obstructive pulmonary disease |
| J44.9 Chronic obstructive pulmonary disease, unspecified |
| J45.0 Predominantly allergic asthma |
| J45.1 Nonallergic asthma |
| J45.9 Asthma, unspecified |
| L40.9 Psoriasis, unspecified |
| M06.9 Rheumatoid arthritis, unspecified |
| M41.9 Scoliosis, unspecified |
| N19 Unspecified kidney failure |
