# Supplementary material for: A cross-sectional investigation of the health needs of asylum seekers in a refugee clinic in Germany
Source: BMC Fam Pract. 2018 May 16;19:64. doi: 10.1186/s12875-018-0758-x (PMC5956552; doi:10.1186/s12875-018-0758-x)
Supplement: Supplementary file 5 — Figure S4. Countries of origin of asylum seekers in Saxony 2015. Countries of origin are plotted with percentage of total asylum seekers who arrived in Saxony between 1 January and 31 December 2015. The data is from Landesdirektion Sachsen, or the State Directorate of Saxony [11]. (DOCX 77 kb) [file 12875_2018_758_MOESM5_ESM.docx]

**Supplement 4**  Frequency of most common diagnoses
